# Buttressing Sutures at the Center of the Mesh May Reduce the Probability of Recurrence in Onlay Incisional Hernia Repair: A Single-Center Study

**DOI:** 10.7759/cureus.56471

**Published:** 2024-03-19

**Authors:** Mümin Demir, Çağrı Akalın

**Affiliations:** 1 General Surgery, Ordu University, Ordu, TUR; 2 General Surgery, Ordu University Training and Research Hospital, Ordu, TUR

**Keywords:** buttressing suture, operative time, chronic pain, recurrence, mesh fixation, incisional hernia

## Abstract

Introduction: An incisional hernia is a common complication after abdominal surgery. Mesh fixation and fascial closure are crucial aspects of repairing incisional hernias. The aim of this study is to investigate the effect of adding a buttressing suture in the center of the mesh in onlay incisional hernia repair on recurrence.

Methods: This retrospective study analyzed 157 patients who underwent elective repair of incisional hernia at Tokat State Hospital between January 2013 and January 2020. The study examined age, gender, comorbidity, smoking, hernia type, defect size, operative time, follow-up time, recurrence, and chronic pain. The patients were divided into two groups based on the type of buttressing suture used: normal and buttressing suture. Recurrence was defined as the reappearance of a hernia at the site of a previous surgery.

Results: The study included 126 patients, with 61 (48.4%) in the control group and 55 (51.6%) in the buttressing suture group. Ten patients reported recurrence, with eight (13.1%) in the control group and two (3.1%) in the buttressing suture group. The difference in recurrence between the groups was statistically significant (p=0.038). The control group had an operative time of 58.79±13.23 minutes, while the buttressing suture group had an operative time of 62.12±13.51 minutes. The statistical analysis did not reveal any significant difference between the two groups (p=0.091). Out of the patients with chronic pain, two (33%) were in the control group and four (66%) were in the buttressing suture group. The incidence of chronic pain did not differ significantly between the two groups (p=0.52).

Conclusion: In incisional hernia repair, the use of buttressing sutures with mesh in incisional hernia repair resulted in decreased recurrence rates without increasing chronic pain or affecting operative time.

## Introduction

An incisional hernia is a common complication that occurs when the abdominal wall weakens at a previous surgical incision, allowing abdominal contents to protrude through the weakened area [1]. In 2015, the European Hernia Society (EHS) issued recommendations for elective midline incisions to reduce hernia risk [2]. The EHS recommends using a continuous suturing technique and advises against using rapidly absorbable sutures to decrease the likelihood of hernia formation. Surgical repair is generally considered the most effective treatment for incisional hernias [3], as it is essential to prevent complications such as pain, bowel obstruction, and the risk of incarceration [4].

In 2023, the EHS guidelines recommended using a mesh-based repair technique for patients with midline incisional hernias [5]. The guidelines also recommended closing the fascial defect without mesh bridging for midline incisional hernia repairs, whether laparoscopic or open. These recommendations were given a Level A. There is no consensus on the type and technique of sutures for fascial closure in mesh-based incisional hernia repair. The use of mesh, whether in an onlay, inlay, or sublay position, has been reported to be effective in reducing recurrence rates [6]. Additionally, to avoid mesh complications such as migration, various techniques are recommended for mesh fixation [7]. However, there is a lack of studies in the literature investigating the impact of adding a buttressing suture to the center of the mesh for reinforcement purposes in onlay incisional hernia repair.

The aim of this study is to investigate the effect of adding a retention suture in the center of the mesh in onlay incisional hernia repair on recurrence.

## Materials and methods

A single-center study was retrospectively designed, and ethics committee approval for the study was obtained from the Clinical Research Ethics Committee of Ordu University, Faculty of Medicine (approval number: 2021/149, date: 17/06/2021). Patients who underwent elective repair of incisional hernia at Tokat State Hospital between January 2013 and January 2020 were analyzed. Patients were divided into two groups according to the buttressing suture used: normal and buttressing suture. Informed consent was obtained from patients prior to surgery.

The study included patients who underwent elective onlay incisional hernia repair and met the following inclusion criteria: (1) over 18 years of age, (2) use of mesh or buttressing suture, and (3) defect size measured by preoperative ultrasound (US). The exclusion criteria were as follows: (1) under 18 years of age, (2) strangulated hernia, (3) history of previous hernia surgery, (4) patients whose information could not be accessed, and (5) death during the follow-up period.

**Figure 1:**
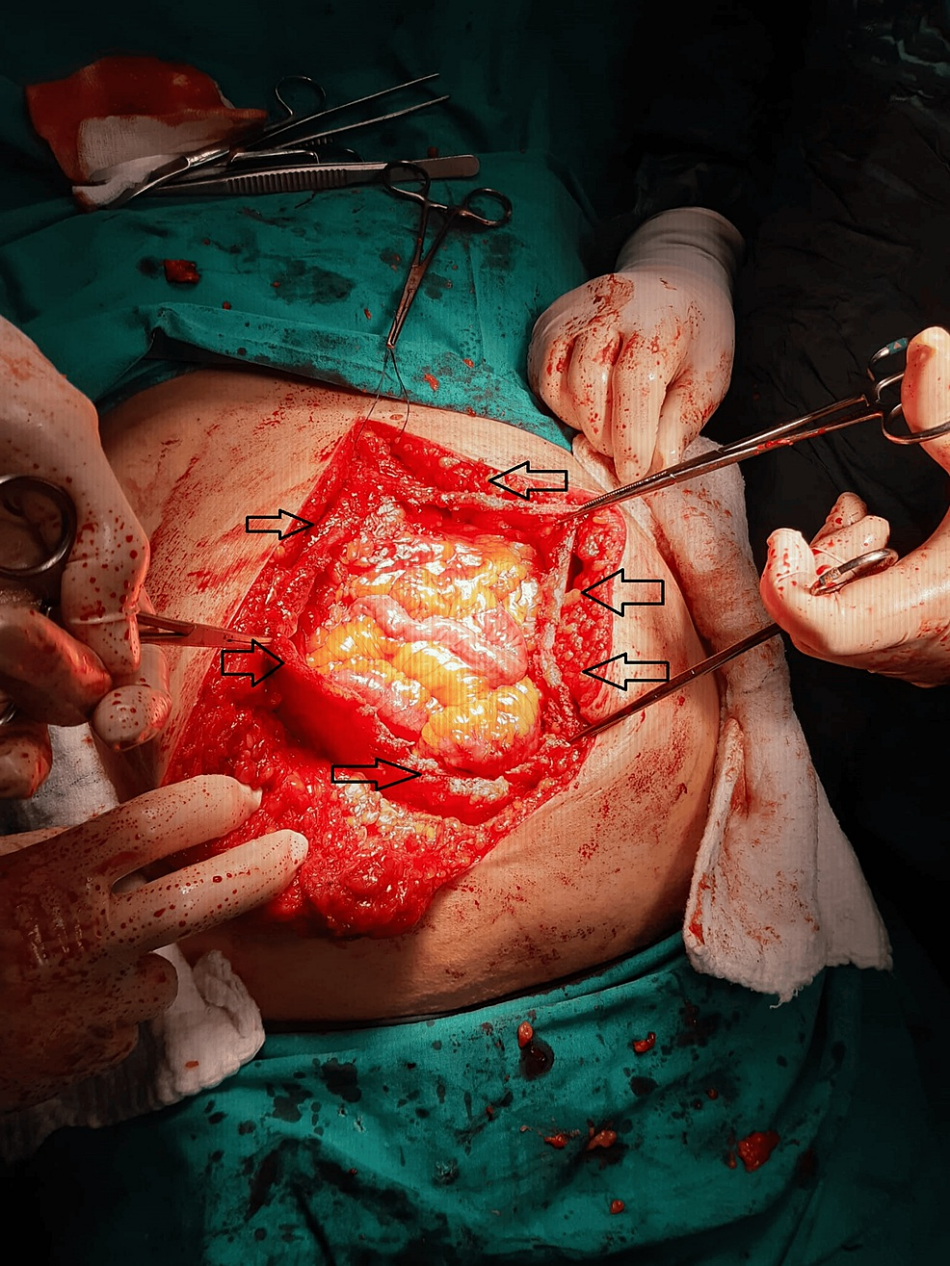
Opening of the hernia sac and separation from adjacent tissue by dissection Arrow signs indicate fascia edges.

**Figure 2:**
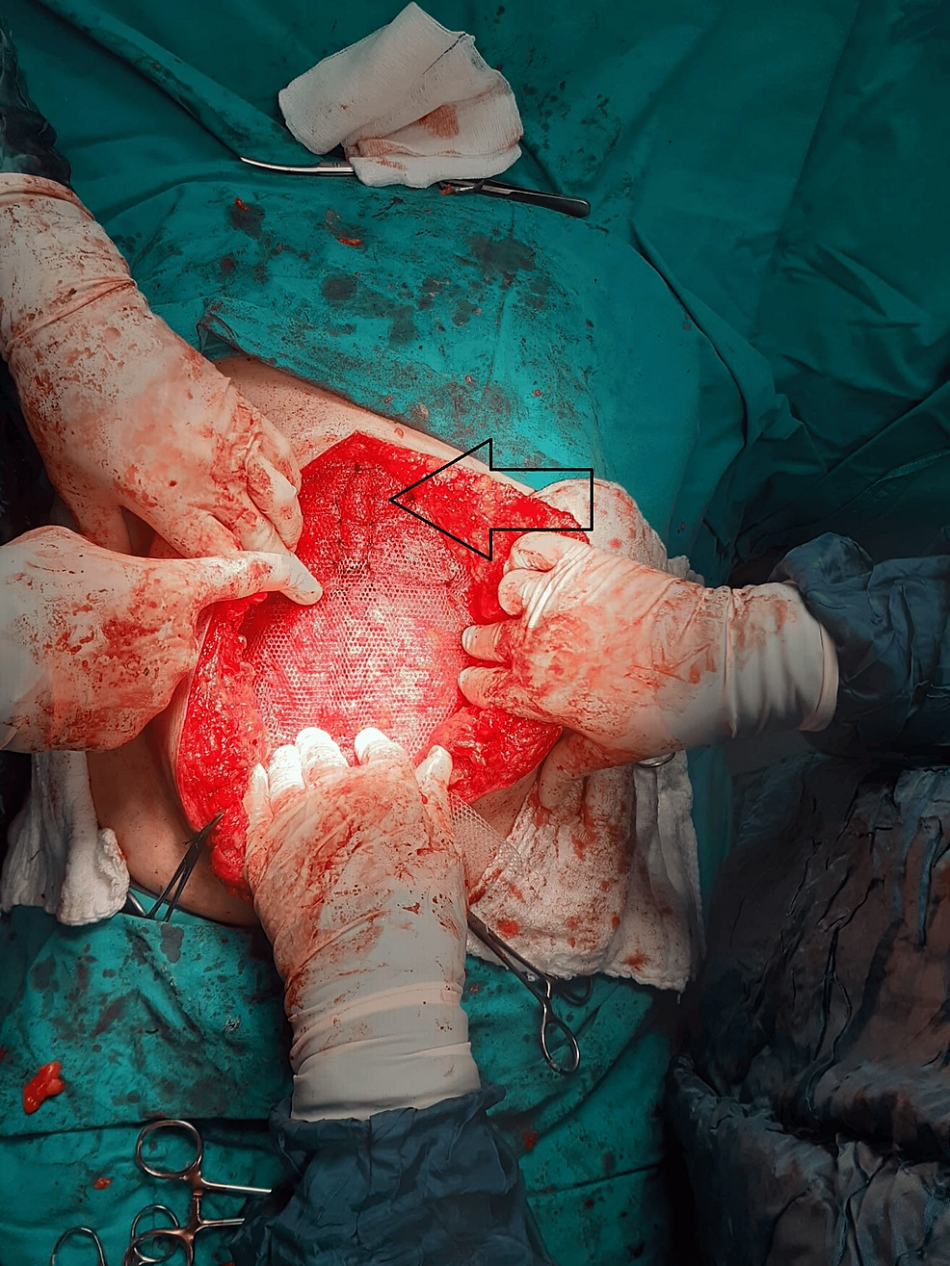
Placement of buttressing sutures on mesh The arrow sign indicates retention sutures.

The surgical operations were performed with the patient under general anesthesia in the supine position by the same surgeon. A single dose of cefazolin sodium (1 g) (Eczacıbaşı, Istanbul, Turkey) was administered intravenously within 30 minutes of the surgical incision. An incision was made at the hernia site, through and under the skin, and the fascial defect was exposed. The hernia sac was opened and separated from the adjacent tissues by dissection (Figure 1). The hernia sac was closed using continuous sutures of 3/0 Vicryl (Polyglactin 910, Ethicon, Ohio, USA). The posterior sheath was sutured with 2/0 Vicryl, and the anterior sheath was sutured with 2/0 polydioxanone suture (PDS) (Ethicon, Amersfoort, Holland). The standard polypropylene mesh was fixed to the anterior sheath with a separated 2/0 polypropylene suture (Prolene, Ethicon, Amersfoort, Holland). The mesh was fixed with buttressing suture (0 PDS) along the facial closure with a continuous fixed suture technique at 2 cm of the facial edge and 2 cm intervals (Figure 2). The operative time was measured from the administration of local anesthesia to skin closure.

The electronic medical records provided the following data: age, gender, comorbidities (including diabetes mellitus, chronic obstructive pulmonary disease, hypertension, and chronic renal failure), smoking status, hernia type, defect size, operative time, follow-up time, recurrence, and chronic pain. The incisional abdominal wall hernias were classified according to the EHS guidelines. The size of the hernia defect was determined by a specialist radiologist using preoperative US as the longest diameter (length or width). Follow-up was conducted by the same surgeon.

Clinical evaluations were performed on all patients at one week, one month, six months, and 12 months. Patients who did not attend the clinical examinations were contacted by phone and invited to the outpatient clinic for the one-year follow-up. All patients were seen at the outpatient clinic during the follow-up period if they presented with signs or symptoms.

Recurrence is defined as the reappearance of a hernia at the site of a previous surgery. It is determined through clinical and US examinations. If patients exhibit symptoms such as pain or swelling, they undergo a clinical examination for recurrence. If the results of the physical examination are suspicious, a specialist radiologist performs a US examination. Pain status is assessed using a visual analog scale (VAS), which rates pain from 0 (no pain) to 10 (worst pain imaginable). Chronic pain is defined as pain persisting in the surgical area for more than six months after surgery, as assessed by the VAS with a score of 3 or higher.

The study presents descriptive statistics for continuous variables as mean and standard deviation (SD) and for categorical variables as numbers and percentages. The distribution of data was assessed using the Kolmogorov-Smirnov test. The relationship between categorical variables was determined using the Chi-square test and Fisher's exact test. The Mann-Whitney U test was used for continuous variables. The statistical software package used for the calculations was the Statistical Package for the Social Sciences (SPSS) for Mac version 24.0 (IBM SPSS Statistics, Armonk, NY). Statistical significance was considered for p-values less than 0.05.

## Results

The study identified 157 patients, of whom 31 could not be contacted for various reasons. Of the remaining 126 patients, 61 (48.4%) were in the control group and 55 (51.6%) were in the buttressing suture group. Table 1 shows no statistically significant differences between the groups in terms of age, gender, comorbidity, smoking, hernia localization, or width.

**Table 1:** Demographic data and hernia classification for individuals The European Hernia Society classification was employed*, with M indicating middle, L indicating lateral, and W indicating width. The data has been represented as N, %, and mean±SD. Percentages are shown in parentheses. Also included is the value at which the p-value is considered significant (p<0.05). DM: diabetes mellitus, COPD: chronic obstructive pulmonary disease, HT: hypertension, CRF: chronic renal failure, N: number, %: percentage, SD: standard deviation

	Control group (N=61)	Buttressing suture group (N=65)	p-value
Age (years)	54.23±11.97	56.89±10.93	0.77
Gender			0.63
Female	26 (42.6)	25 (38.5)	
Male	35 (57.4)	40 (61.5)	
Comorbidities			0.84
DM	19 (31.1)	17 (26.1)	
COPD	8 (13.1)	9 (13.8)	
HT	24 (39.3)	26 (40)	
CRF	1 (1.6)	1 (1.5)	
Smoking	7 (11.4)	12 (18.4)	0.27
Hernia localization*			0.73
M1 (subxiphoidal)	2 (0.3)	1 (1.5)	
M2 (epigastric)	21 (34)	24 (36.9)	
M3 (umbilical)	26 (42.6)	23 (35.4)	
M4 (infraumbilical)	8 (1.2)	13 (20)	
M5 (suprapubic)	1 (0.1)	2 (3)	
L1 (subcostal)	3 (0.3)	2 (3)	
L2 (flank)	-	-	
L3 (iliac)	-	-	
L4 (lumbar)	-	-	
Hernia width*			0.98
W1	29 (47.5)	31 (47.7)	
W2	32 (52.4)	34 (52.3)	

A total of 10 patients reported recurrence, eight (13.1%) in the control group and two (3.1%) in the buttressing suture group. The difference between the groups in terms of recurrence was statistically significant (p=0.038). There were no significant differences in age, gender, comorbidity, and smoking between patients with and without recurrence (p=0.16, p=0.62, p=0.24, and p=0.46, respectively). The distribution of hernia locations in patients with recurrence (n=10) was as follows: M2, 3 (30%); M3, 5 (50%); M4, 1 (10%); and L1, 1 (10%). There was no statistically significant difference found between recurrence and hernia location (p=0.88) or between the recurrence rate and hernia width (p=0.75).

The operative time for all patients was 60.51±13.45 minutes. The control group had an operative time of 58.79±13.23 minutes, while the buttressing suture group had an operative time of 62.12±13.51 minutes. There was no statistically significant difference between the groups (p=0.091). The operative time for hernia width W1 was 54.05±7.52 minutes, while for W2, it was 66.38±14.89 minutes. There was a statistically significant difference between the two (p<0.001).

Out of the total number of patients, 4.7% (n=6) reported experiencing chronic pain. Statistical analysis showed no significant differences between patients with chronic pain and those without in terms of age, gender, comorbidity, and smoking (p=0.48, p=0.71, p=0.34, and p=0.86, respectively). Among the patients with chronic pain (n=6), two were in the control group (33%) and four were in the buttressing suture group (66%). However, there was no significant difference in the incidence of chronic pain between the two groups (p=0.52). The patients with chronic pain (n=6) were distributed according to hernia localization as follows: 33% (n=2) had M2, 50% (n=3) had M3, and 17% (n=1) had L1. Hernia width was classified as W1 for 33% (n=2) of patients and W2 for 66% (n=4) of patients. There was no statistically significant difference between chronic pain and hernia localization or width (p=0.67 and p=0.61, respectively).

The patients were followed up for an average of 49.1±23.6 months. The control group was followed up for an average of 52.3±25.7 months, while the buttressing group was followed up for an average of 46.5±22.8 months. There was no significant difference in follow-up time between the two groups (p=0.88). The control group had a time to recurrence of 27.7 months, while the buttressing suture group had a time to recurrence of 39.4 months.

## Discussion

In this study, buttressing sutures were added to the center of the mesh in addition to the fixation sutures in onlay incisional mesh repair. The study found that buttressing sutures to the center of the mesh, in addition to fixation sutures, statistically reduced recurrence rates. The potential for buttressing sutures to cause chronic pain was also evaluated, and no statistically significant evidence of such an effect was found. Additionally, we observed that the buttress suture technique did not have a statistically significant negative impact on operative time.

The EHS guidelines for midline incisional hernia discuss the possibility of performing fascial closure without requiring myofascial release [5]. Two cross-sectional studies have evaluated the impact of hernia defect width on fascial closure. Love et al. found that patients who required myofascial release had an average hernia width of 12.78 cm, compared to 7.53 cm for those who did not require it, with significant differences (p<0.001) [8]. Blair et al. reported that the former group had an average hernia width of 11.5 cm, while the latter had an average of 7.6 cm (p=0.002) [9]. They also found that a hernia width over 8.3 cm significantly predicts the need for myofascial release (AUC=0.72). The authors measured hernia defects using computed tomography (CT). In our study, while all patients underwent US examination, some did not undergo CT examination. Therefore, we used US examination as the basis for evaluating hernia defects in the study. According to the 2015 EHS guideline, CT is the most accurate radiological examination for incisional hernia examination, followed by US, which also has good accuracy [10,11]. In this study, patients who underwent myofascial release surgery were not included, and patients with W3 (>10 cm) incisional hernias were excluded. In our study, we identified subgroups based on midline/lateral and W1/W2; we think that the exclusion criterion may have contributed to the homogeneity of our study.

Recurrence is a common complication after surgery. According to the 2023 EHS Guidelines, mesh placement in the retromuscular plane is recommended for patients with midline incisional hernias to reduce the risk of recurrence [5]. This recommendation is supported by Level A evidence. In our study, we applied mesh-based repair to all patients with midline and lateral hernias. Unfortunately, we were unable to conduct the recommended retromuscular repair due to the surgeon's lack of experience. However, the surgeon performed the onlay technique recommended by the EHS's Good Practice Statement Level A, which requires proficiency in various mesh positioning techniques, including onlay, retromuscular, and intraperitoneal.

The updated guidelines for abdominal closure recommend using a continuous small bites suturing technique with a slowly absorbable suture for the closure of elective midline incisions. This recommendation is based on three randomized controlled trials (RCTs) published since the 2015 guidelines [12-14]. It is suggested to use a slowly absorbable monofilament suture in a single-layer aponeurotic closure technique without separately closing the peritoneum. The current recommended method of fascial closure is a small bites technique with a suture-to-wound length (SL/WL) ratio of at least 4/1. It is important to maintain a balanced and objective approach when evaluating the evidence. The authors of these guidelines included a statement on abdominal wall closure as a surgical risk factor for developing an incisional hernia, despite the low quality of evidence and weak strength of recommendation. In this study, the anterior and posterior fascia were closed with absorbable sutures. Additionally, a buttressing suture was applied over the mesh along the fascial edges in a continuous manner using a PDS after closing the anterior fascia. Therefore, it is important not to confuse the applied suture technique with the retention suture, which some surgeons particularly use in acute settings to deal with a burst abdomen and/or to achieve abdominal wall closure. In this study, we believe that buttressing sutures positively support facial closure even if they only pass through the anterior fascia.

Chronic pain after herniorrhaphy is a significant concern. Studies suggest that the type of mesh or fixation method used may influence the development of chronic pain after surgery [15]. However, the presence of the prosthetic mesh itself may not significantly contribute to postoperative complaints [16]. It is also important to note that the occurrence of chronic pain can be affected by the mesh fixation technique, including the type of mesh or fixation method used [17]. Our study found no significant difference in chronic pain between the two groups. This suggests that placing buttressing sutures solely in the midline may have positively impacted chronic pain outcomes by not affecting the peripheral parts of the mesh. However, we acknowledge that the lack of a uniform patient group, varying hernia locations and sizes, and the small sample size might have influenced this result, highlighting the potential for a type 2 error in outcome analysis.

The study has several limitations. These include its retrospective design, lack of randomization, small sample size, heterogeneity, and short follow-up period. We acknowledge that the retrospective design of our study and the lack of a random method in patient selection are major shortcomings. Patient selection was determined solely by the surgeon's preference, without relying on predetermined criteria. This may lead to significant bias in group assignments and may have implications for the generalization of the study's results. By stating this methodological limitation clearly, we emphasize the need for caution when interpreting and generalizing our findings. Additionally, it was not possible to evaluate patients' postoperative pain and quality of life scales. Furthermore, there are concerns about the objectivity of the responses as communication with patients regarding chronic pain was conducted via telephone. Although our operation technique produced positive results in the study, it still requires further development to become standardized. However, our study has several positive aspects. For instance, to the best of our knowledge, it is the first study in the literature to place buttress sutures in the center of the mesh. Additionally, the study was conducted by the same surgeon at a single center, which is considered an advantage.

## Conclusions

In conclusion, this study provides evidence supporting the efficacy of buttressing sutures to the center of the mesh in onlay incisional hernia repair. The findings demonstrate a reduction in recurrence rates, suggesting an improved surgical outcome without significantly affecting operative times or increasing the risk of chronic pain. However, it is important to acknowledge the limitations of the study's retrospective design, including the small sample size, heterogeneity, and short follow-up period. Despite these limitations, the study's outcomes are promising and require further investigation through prospective, randomized trials with larger cohorts. Such future studies are essential to validate the findings, refine surgical practices, and ultimately improve patient care in the field of incisional hernia repair.
